# The Test-Retest Reliability of Anatomical Co-Ordinate Axes Definition for the Quantification of Lower Extremity Kinematics During Running

**DOI:** 10.2478/v10078-012-0075-8

**Published:** 2012-12-30

**Authors:** Jonathan Sinclair, Paul John Taylor, Andrew Greenhalgh, Christopher James Edmundson, Darrell Brooks, Sarah Jane Hobbs

**Affiliations:** 1 Division of Sport, Exercise and Nutritional Sciences, University of Central Lancashire.; 2 School of Psychology, University of Central Lancashire.; 3 School of Life Sciences, University of Hertfordshire.; 4 Faculty of Health, University of Staffordshire.

**Keywords:** Gait analysis, running, kinematics, repeatability, motion analysis

## Abstract

Three-dimensional (3-D) kinematic analyses are used widely in both sport and clinical examinations. However, this procedure depends on reliable palpation of anatomical landmarks and mal-positioning of markers between sessions may result in improperly defined segment co-ordinate system axes which will produce in-consistent joint rotations. This had led some to question the efficacy of this technique. The aim of the current investigation was to assess the reliability of the anatomical frame definition when quantifying 3-D kinematics of the lower extremities during running. Ten participants completed five successful running trials at 4.0 m·s^−1^ ± 5%. 3-D angular joint kinematics parameters from the hip, knee and ankle were collected using an eight camera motion analysis system. Two static calibration trials were captured. The first (test) was conducted prior to the running trials following which anatomical landmarks were removed. The second was obtained following completion of the running trials where anatomical landmarks were re-positioned (retest). Paired samples t-tests were used to compare 3-D kinematic parameters quantified using the two static trials, and intraclass correlations were employed to examine the similarities between the sagittal, coronal and transverse plane waveforms. The results indicate that no significant (p>0.05) differences were found between test and retest 3-D kinematic parameters and strong (R^2^≥0.87) correlations were observed between test and retest waveforms. Based on the results obtained from this investigation, it appears that the anatomical co-ordinate axes of the lower extremities can be defined reliably thus confirming the efficacy of studies using this technique.

## Introduction

Three-dimensional (3-D) kinematic analyses are used widely in both sport and clinical examinations. The computer aided movement analysis in a rehabilitation group (Leo, 1995) proposed recommendations for anatomical landmarks used to define the anatomical frame of the lower extremities. This was borne out of the work by Cappozzo et al. (1995) and was designed to increase the efficacy of future studies in modelling lower extremity segments.

The calibrated anatomical systems technique (CAST) offers the ability to model each body segment in six degrees of freedom (Cappozzo et al., 1995). The CAST technique involves the quantification of an anatomical coordinate system axes for each segment via the identification of anatomical landmarks through external palpation which is then calibrated with respect to corresponding arrays of technical tracking clusters (Richards and Thewlis, 2008). This technique is currently considered to be the gold standard for 3-D kinematic analyses (Richards and Thewlis, 2008; Sinclair et al., 2012). However, anatomical landmark identification by manual palpation and corresponding marker placement is not an error-free technique, and mal-positioning of anatomical landmarks may cause improperly defined segment co-ordinate system axes which will result in erroneous joint rotations (Kabada et al., 1989; Ferber et al., 2002). Analyses using 3-D motion capture systems are now common place in biomechanics research and reliability is of paramount importance, particularly in epidemiological or aetiological analyses when clinical decisions are made.

In sport and clinical research, where multiple participants are examined or patient’s gait must be assessed over time, it is essential to ensure that the identification of the relevant joint centres is reproducible. Reliable segment coordinate system axes are important as they provide reliable and consistent movement interpretation. Kadaba et al. (1989) and Della Croce et al. (1999) suggest that even small differences in the orientation and placement of markers forming the segment co-ordinate system can lead to sizeable differences in the calculation of joint angular parameters which may in turn inhibit the interpretation of the collected data.

Therefore analyses utilizing 3-D motion capture techniques clearly necessitate the accurate palpation of anatomical landmarks to produce repeatable, segmental anatomical co-ordinate systems (Della Croce et al., 2005). However, Della Croce et al. (2005) suggest it is difficult to place anatomical markers in exactly the same location and the determination of their location lacks accuracy and precision. Previous investigations have been conducted examining the reliability of 3-D kinematic techniques (McGinley et al., 2009; Rothstein and Echternach, 1993; Pohl et al., 2010); however, the majority of these have examined either inter-session or inter-assessor reliability between sessions. Whilst these factors are clearly important to the efficacy of 3-D kinematic protocols they do not allow the reliability of anatomical frame definition to be examined effectively as different (inter-session) dynamic data is being applied to the static anatomical reference trials obtained from each session. Therefore, despite the number of investigations utilizing 3-D analysis, there is currently a paucity of research investigating the true test-retest reliability in defining the segment anatomical coordinate system and the influence that differences in anatomical frame definition may have on the 3-D kinematic parameters measured during the stance phase of running.

The aim of the current investigation is therefore to assess the reliability of the anatomical frame definition when quantifying 3-D kinematics of the lower extremities during running.

## Methods

### Participants

Ten participants (7 males and 3 females) volunteered to take part in this investigation (age 22.4 ± 2.05 years; body height 179.4 ± 6.2 cm; body mass 79.1 ± 8.2 kg; shoe size 7–9 UK). All were injury free at the time of data collection and provided written informed consent in accordance with the declaration of Helsinki. Ethical approval for this project was obtained from the University of Central Lancashire School of Psychology ethics committee.

### Procedures

An eight camera motion analysis system (Qualisys^™^ Medical AB, Goteburg, Sweden) captured kinematic data at 250 Hz and was calibrated before each data collection session. Participants ran at 4.0m/s (±5%) over a force platform (Kistler, Kistler Instruments Ltd.,) sampling at 1000 Hz, stance time was determined as the time over which 20 N or greater of vertical force was applied to the force platform (Sinclair et al., 2011). Velocity was controlled using infrared photocells Newtest 300 (Newtest, Oy Koulukatu 31 B 11 90100 Oulu Finland).

**Figure 1:**
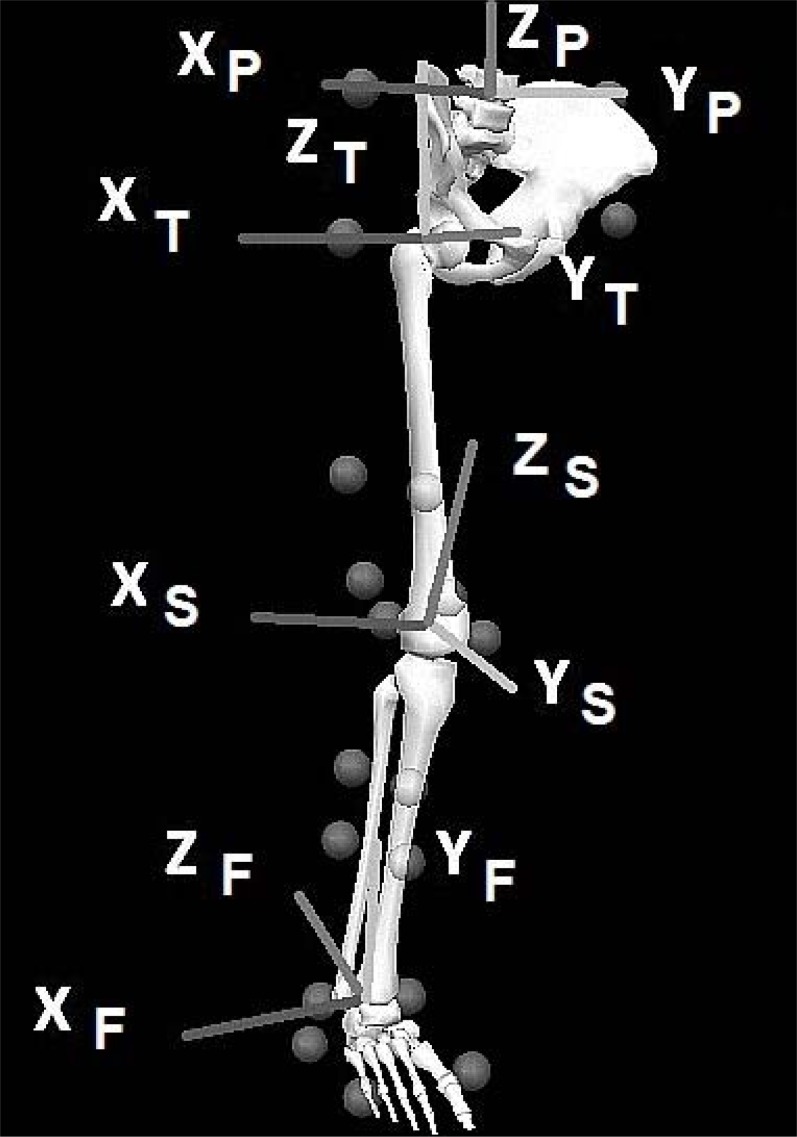
Pelvic, thigh, tibial and foot segments, with segment co-ordinate system axes. (P= Pelvis, S= Shank, T= tibia and F = foot)

The marker configuration utilized for the study to record lower limb kinematics was based on the CAST technique (Cappozzo et al., 1995). In order to define the anatomical reference frames of the pelvis, thigh, foot and shank segments; retro-reflective markers were attached to the 1st and 5th metatarsal heads, medial and lateral malleoli, medial and lateral epicondyle of the femur, greater trochanter of the right leg, iliac crest, anterior superior iliac spines and posterior superior iliac spines (Figure 1). Hip joint centre was determined based on the Bell et al. (1989) equations via the positions of the PSIS and ASIS markers.

**Figure 2:**
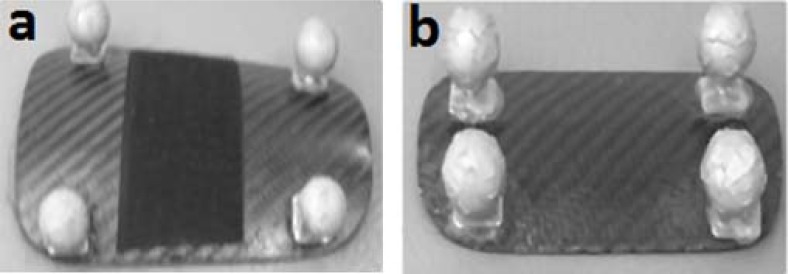
Carbon fiber tracking clusters as positioned on the a. thigh and shank and b. pelvic segments

Two static calibration trials were captured with the participant standing in the anatomical position. The first static (test) was conducted prior to the running trials and the anatomical landmarks were removed. Following completion of the running trials the anatomical landmarks were re-positioned and the second static trial (retest) was obtained. Cluster markers used to define the technical tracking frame of each segment remained rigidly in place for the duration of the analysis and were not removed, allowing the test-retest reliability of the anatomical frame to be examined. The tracking clusters positioned on the pelvis, thigh and shank were comprised of four 19mm spherical reflective markers mounted to a thin sheath of lightweight carbon fiber (Figure 2) with a length to width ratio of 1.5-1, in accordance with the Cappozzo et al. (1997) recommendations. The technical frame of the foot segment was defined using four retro-reflective markers glued rigidly onto the footwear (Saucony pro grid guide 2, sizes 7–9 UK). The same model of footwear was used for all participants and was selected to represent typical running footwear.

### Data processing

Motion files from each participant were applied to both static trials. Kinematic parameters from static one (Test) and two (Retest) were quantified using Visual 3-D (C-Motion Inc, Germantown, USA) and filtered at 10 Hz using a zero-lag low pass Butterworth 4^th^ order filter. This was selected as being the frequency at which 95% of the signal power was below following a fast fourier transform (FFT) using Labview software (National instruments, Austin TX). Lower extremity joint angles were created using an XYZ cardan sequence of rotations (Sinclair et al., 2012). All data were normalized to 100% of the stance phase, then mean processed gait trial data was reported. 3-D kinematic measures from the hip, knee and ankle which were extracted for statistical analysis were 1) angle at footstrike, 2) angle at toe-off, 3) range of motion from footstrike to toe-off during stance, 4) peak angle during stance, 5) peak angular excursion from footstrike to peak angle 6) velocity at footstrike, 7) velocity at toe-off and 8) peak velocity.

### Analysis

Descriptive statistics including means and standard deviations were calculated for each condition. Differences in stance phase kinematic parameters were examined using paired samples t-tests with significance accepted at the p≤0.05 level. The Shapiro-wilk statistic for each condition confirmed that the data were normally distributed. Intra-class correlations were utilized to compare test and retest sagittal, coronal and transverse plane waveforms of the hip, knee and ankle. All statistical procedures were conducted using SPSS 19.0 (SPSS Inc, Chicago, USA).

## Results

### Joint Angles

**Figure 3:**
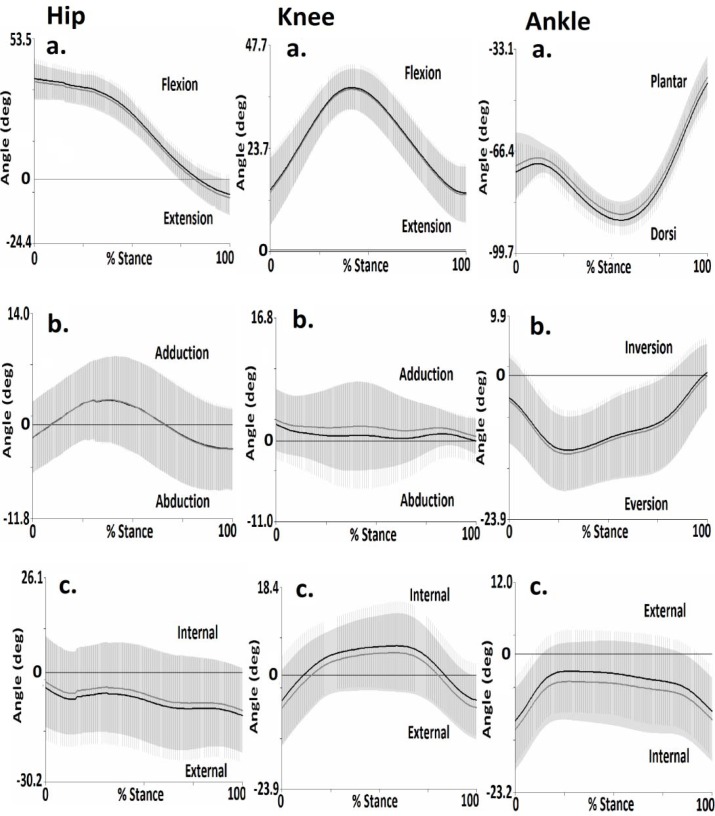
Mean and standard deviation hip, knee and ankle joint kinematics in the a. sagittal, b. coronal and c. transverse planes for Test (black line) and Retest (grey line), running (shaded area is 1 ±SD, Test = grey shade and Retest = horizontal).

Figure 3 presents the mean and standard deviation 3-D angular kinematic waveforms from of the lower extremities during the stance phase. Tables 1–3 present 3-D joint angles obtained as a function of test and retest static trials.

#### Hip

The results indicate that no significant (p>0.05) differences in hip joint kinematics in the sagittal, coronal and transverse planes were observed between test and retest parameters.

#### Knee

The results indicate that no significant (p>0.05) differences in knee joint kinematics in the sagittal, coronal and transverse planes were observed between test and retest parameters.

#### Ankle

The results indicate that no significant (p>0.05) differences in ankle joint kinematics in the sagittal, coronal and transverse planes were observed between test and retest parameters.

Comparisons between pre and post kinematic waveforms for the hip joint revealed strong correlations for the sagittal (R^2^= 0.99), coronal (R^2^=0.98) and transverse (R^2^= 0.96) planes. For the knee joint strong correlations were observed in the sagittal (R^2^= 0.99), coronal (R^2^=0.96) and transverse (R^2^= 0.96) planes. For the ankle joint strong correlations were observed in sagittal (R^2^= 0.96), coronal (R^2^=0.90) and transverse (R^2^= 0.91) planes.

### Joint Velocities

**Figure 4:**
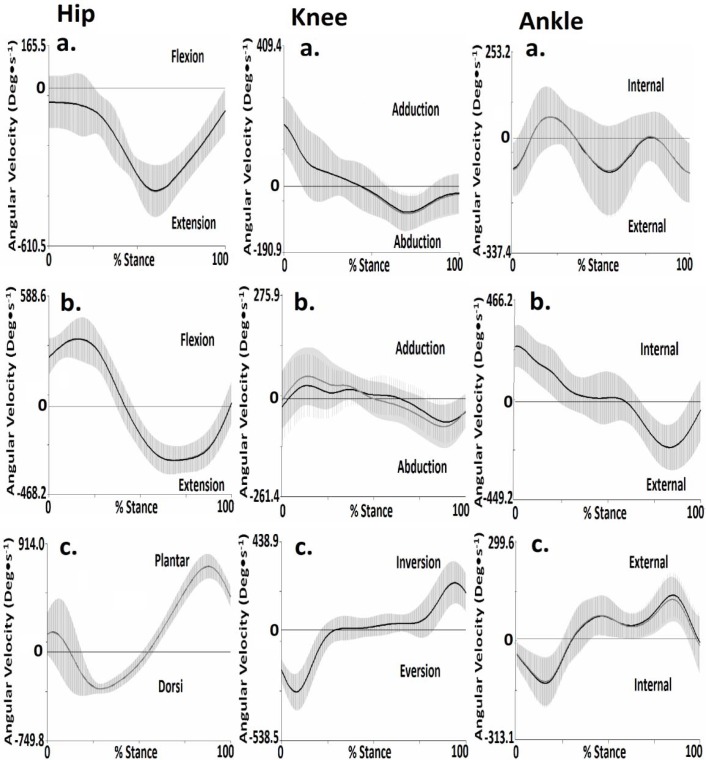
Mean and standard deviation hip, knee and ankle joint velocities in the a. sagittal, b. coronal and c. transverse planes for Test (black line) and Retest (grey line), running (shaded area is 1 ±SD, Test = grey shade and Retest = horizontal)

Figure 4 presents the mean and standard deviation 3-D angular kinematic waveforms from of the lower extremities during the stance phase. Tables 4–6 present 3-D joint velocities obtained as a function of test and retest static trials.

#### Hip

The results indicate that no significant (p>0.05) differences in hip joint velocities in the sagittal, coronal and transverse planes were observed between test and retest parameters.

#### Knee

The results indicate that no significant (p>0.05) differences in knee joint velocities in the sagittal, coronal and transverse planes were observed between test and retest parameters.

#### Ankle

The results indicate that no significant (p>0.05) differences in ankle joint velocities in the sagittal, coronal and transverse planes were observed between test and retest parameters.

Comparisons between pre and post kinematic waveforms for the hip joint revealed strong correlations for the sagittal (R^2^= 0.99), coronal (R^2^=0.99) and transverse (R^2^= 0.97) planes. For the knee joint strong correlations were observed in the sagittal (R^2^=0.99), coronal (R^2^=0.99 and transverse R^2^= 0.92 planes. For the ankle joint strong correlations were observed in sagittal R^2^= 0.92, coronal R^2^=0.90 and transverse R^2^= 0.87 planes.

## Discussion

The aim of the current investigation was to determine the test-retest reliability of the segment anatomical reference frame definition. In the present study, running trials were analysed simultaneously using two different anatomical coordinate systems. This represents the first study investigating the test-retest reliability in defining the lower extremity segment anatomical coordinate system axes and their potential influence on 3-D kinematic parameters during the stance phase of running.

The major finding from the current investigation is that the different anatomical reference frames obtained from the test and retest static trials had no significant (p>0.05) effect on 3-D kinematic parameters. This opposes the findings of Kabada et al. (1989) who observed that the angular deviations when examining reliability were much greater than those observed in the current study.

It is beyond the latitude of this study to specify acceptable levels of consistency for 3-D kinematic information. However, in their review paper, McGinley et al. (2009) propose that in most common clinical situations errors of 2° or less are highly likely to be considered acceptable and errors of between 2 and 5° are also likely to be regarded as reasonable. It is proposed that angular deviations in excess of 5° should be construed as excessive as they may be sufficient to misinform clinical analyses. Based on these recommendations it appears that the technique utilized in the current investigation is associated with low levels of error as the majority of test-retest angular deviations were found to be < 2°.

The intra class correlation analyses indicate that stance phase kinematic waveforms in the sagittal plane exhibited very good agreement (R^2^≥0.92) between test and retest defined coordinate axes. Furthermore, whilst coronal and transverse plane waveforms also exhibited good agreement the conformity (R^2^ ≥0.87) was lower than those observed in the sagittal plane. This concurs with the findings of Kabada et al. (1989) who noted that coronal and transverse plane angles were affected more pointedly than the sagittal plane profiles by differences in anatomical frame axes definition.

The lowest correlations between test and retest waveforms were observed for ankle joint parameters in all three anatomical planes. It is proposed that this relates to the fact that the anatomical co-ordinate system axes of the foot were defined by placing markers directly onto the shoe which has been identified as problematic. This is because it is more difficult to palpate non visible landmarks through the shoe. Furthermore, there is almost a certain movement of the foot within the shoe (Stacoff et al., 1992), thus it is questionable as to whether anatomical markers located on the shoe provide comparable results to those placed on the foot itself. Future studies may wish to re-examine the reliability of anatomical frame definition when placing markers directly onto the foot.

With the aim of increasing the efficacy and reliability of 3-D kinematic data, researchers have also developed methods of quantifying segmental axes of rotation that are independent of anatomical landmarks. The most common is the functional method of identifying segmental parameters has been proposed as an effective way to reduce the proposed variability of anatomical definitions (Besier et al., 2003; Della Croce et al., 1999). However, the use of markerless technology to record 3-D kinematics is still a minority technique (Richards and Thewlis, 2008) and has been limited by the intricacy of obtaining precise 3-D kinematics using this approach (Corazza et al., 2006). Future research may wish to replicate the current investigation using markerless anatomical frame definition to further examine the efficacy of this technique.

The fact that this paper focused solely on 3-D angulation and angular velocities is potentially a limitation of the current investigation. Future investigations should focus on additional kinetic parameters such as joint moments which may be influenced by differences in anatomical frame definition (Thewlis et al., 2008). Joint moments have strong sporting and clinical significance and may also be influenced by variations in the anatomical frame thus it is important to also consider their reliability. Finally, care should be taken when attempting to generalize the findings of this study to investigations examining pathological kinematics. It is likely that variations will exist in the relative contributions of the sources of measurement error in participants who exhibit an abnormal gait pattern (Gorton et al., 2009). For participants with skeletal alignment pathologies, palpation and subsequent marker placement may be more complex and result in reduced reliability (Gorton et al., 2009).

In conclusion, based on the results obtained from the methodologies used in the current investigation, it appears that the anatomical co-ordinate axes of the lower extremities can be defined reliably. Future research should focus on the efficacy and advancement of markerless techniques.

## Figures and Tables

**Table 1:** Hip joint kinematics (means, standard deviations) from the stance limb as a function of Test and Retest anatomical co-ordinate axes (* = Significant main effect p≤0.05)

	**Test**	**Retest**	**Mean difference (°)**
**Hip**			
**X (+ = flexion/ − = extension)**			
Angle at Footstrike (°)	38.21 ± 3.96	39.11 ± 6.43	0.9
Angle at Toe-off (°)	−5.56 ± 6.77	−4.66 ± 6.69	0.9
Range of Motion (°)	43.77 ± 5.91	43.58 ± 6.06	0.19
Relative Range of Motion (°)	0.96 ± 0.97	0.94 ± 0.98	0.02
Peak Flexion (°)	38.73 ± 5.16	40.71 ± 5.12	1.98
**Y (+ =adduction/−=abduction)**			
Angle at Footstrike (°)	−2.02 ± 3.96	−2.55 ± 4.84	0.53
Angle at Toe-off (°)	−3.82 ± 4.65	−4.40 ± 4.63	0.58
Range of Motion (°)	4.51 ± 2.17	5.01 ± 3.63	0.5
Relative Range of Motion (°)	5.34 ± 3.16	5.38 ± 3.19	0.02
Peak Adduction (°)	3.38 ± 4.90	2.94 ± 5.06	0.44
**Z (+=internal /− =external)**			
Angle at Footstrike (°)	−5.34 ± 11.36	−7.01 ± 11.71	1.67
Angle at Toe-off (°)	−13.42 ± 10.54	−13.58 ± 11.10	0.16
Range of Motion (°)	43.77 ± 5.91	43.58 ± 6.06	0.19
Relative Range of Motion (°)	9.53 ± 3.86	9.70 ± 3.78	0.17
Peak External rotation (°)	−13.99 ± 9.08	−15.16 ± 10.20	1.67

**Table 2:** Knee joint kinematics (means, standard deviations) from the stance limb as a function of Test and Retest anatomical co-ordinate axes (* = Significant main effect p≤0.05).

	**Test**	**Retest**	**Mean difference (°)**
**Knee**			
**X (+ = flexion/ − = extension)**			
Angle at Footstrike (°)	13.88 ± 6.52	14.27 ± 6.72	0.39
Angle at Toe-off (°)	12.99 ± 5.32	13.45 ± 5.92	0.46
Range of Motion (°)	5.67 ± 2.63	5.70 ± 2.56	0.03
Relative Range of Motion (°)	24.70 ± 4.45	24.46 ± 4.44	0.24
Peak Flexion (°)	38.24 ± 3.56	38.87 ± 4.42	0.63
**Y (+ =adduction/−=abduction)**			
Angle at Footstrike (°)	3.33 ± 4.07	2.92 ± 4.03	0.41
Angle at Toe-off (°)	0.89 ± 2.75	0.10 ± 2.91	0.79
Range of Motion (°)	3.58 ± 2.70	3.56 ± 2.70	0.02
Relative Range of Motion (°)	5.04 ± 2.87	5.83 ± 3.22	0.79
Peak Adduction (°)	−1.86 ± 4.11	−2.52 ± 4.40	0.66
**Z (+ =internal/− =external)**			
Angle at Footstrike (°)	−5.08 ± 4.87	−2.89 ± 6.29	2.19
Angle at Toe-off (°)	−5.56 ± 6.77	−4.46 ±6.69	1.1
Range of Motion (°)	3.32 ± 1.50	3.35 ± 1.53	0.03
Relative Range of Motion (°)	12.97 ± 3.72	12.60 ± 3.82	0.37
Peak Internal Rotation (°)	8.46 ± 5.18	10.42 ± 5.96	1.96

**Table 3:** Ankle joint kinematics (means, standard deviations) from the stance limb as a function of Test and Retest anatomical co-ordinate axes (* = Significant main effect p≤0.05)

	**Test**	**Retest**	**Mean difference (°)**
**Ankle**			
**X (+ =plantar/− =dorsi)**			
Angle at Footstrike (°)	−72.48 ± 11.10	−73.64 ± 10.34	1.16
Angle at Toe-off (°)	−43.44 ± 3.91	−45.16 ± 3.87	1.72
Range of Motion (°)	28.47 ± 12.67	28.48 ± 12.60	0.01
Relative Range of Motion (°)	16.35 ± 11.45	16.44 ± 11.53	0.09
Peak Dorsiflexion (°)	−87.35 ± 3.84	−89.99 ± 4.55	2.64
**Y (+ =inversion/ − =eversion)**			
Angle at Footstrike (°)	−3.72 ± 7.41	−3.05 ± 7.70	0.67
Angle at Toe-off (°)	0.25 ± 4.97	1.13 ± 5.38	0.88
Range of Motion (°)	5.34 ± 2.22	5.43 ± 2.36	0.09
Relative Range of Motion (°)	9.51 ± 3.38	9.28 ± 3.39	0.23
Peak Eversion (°)	−13.24 ± 6.65	−12.33 ± 6.94	0.91
**Z (− =internal/+ =external)**			
Angle at Footstrike (°)	−12.13 ± 6.97	−9.91 ± 6.71	2.22
Angle at Toe-off (°)	−10.42 ± 7.17	−8.30 ± 7.26	2.12
Range of Motion (°)	2.08 ± 1.47	2.43 ± 2.36	0.35
Relative Range of Motion (°)	9.39 ± 3.57	9.66 ± 3.54	0.27
Peak Internal Rotation (°)	−2.75 ± 7.63	−0.22 ± 7.17	2.53

**Table 4:** Hip joint velocities (means, standard deviations) from the stance limb as a function of Test and Retest anatomical co-ordinate axes (* = Significant main effect p≤0.05)

	**Test**	**Retest**	**Mean difference (Deg.s^−1^)**
**Hip**			
**X (+ = flexion/ − = extension)**			
Velocity at FootStrike (Deg.S^−1^)	−54.03 ± 95.74	−55.75 ± 94.68	1.72
Velocity at Toe-Off (Deg.S^−1^)	−93.65 ± 76.21	92.19 ± 79.21	1.46
Peak Extension Velocity (Deg.S^−1^)	−419.36 ± 94.91	−417.73 ± 94.25	1.63
**Y (+ =adduction/−=abduction)**			
Velocity at FootStrike (Deg.S^−1^)	182.88 ± 66.48	183.37 ± 65.84	0.49
Velocity at Toe-Off (Deg.S^−1^)	−21.24 ±58.69	−18.43 ± 58.72	2.81
Peak Abduction Velocity (Deg.S^−1^)	−107.25 ± 36.60	−102.49 ± 38.37	5.26
**Z (+=internal /− =external)**			
Velocity at FootStrike (Deg.S^−1^)	−94.03 ± 67.55	−90.65 ± 76.32	3.38
Velocity at Toe-Off (Deg.S^−1^)	−102.24 ± 68.22	−101.20 ± 68.62	1.04
Peak Internal Rotation Velocity (Deg.S^−1^)	120.46 ± 42.87	120.60 ± 43.87	0.14

**Table 5:** Knee joint velocities (means, standard deviations) from the stance limb as a function of Test and Retest anatomical co-ordinate axes (* = Significant main effect p≤0.05)

	**Test**	**Retest**	**Mean difference (Deg.s^−1^)**
**Knee**			
**X (+ = flexion/ − = extension)**			
Velocity at FootStrike (Deg.S^−1^)	265.89 ± 89.78	263.81 ± 83.88	2.08
Velocity at Toe-Off (Deg.S^−1^)	20.05 ± 76.63	16.86 ± 76.64	3.19
Peak Flexion Velocity (Deg.S^−1^)	397.68 ± 39.85	397.08 ± 61.33	0.6
Peak Extension Velocity (Deg.S^−1^)	−320.42 ± 59.76	−322.36 ± 59.76	1.94
**Y (+ =adduction/−=abduction)**			
Velocity at FootStrike (Deg.S^−1^)	−13.67 ± 62.60	−21.57 ± 75.60	7.9
Velocity at Toe-Off (Deg.S^−1^)	−34.25 ± 30.66	−36.44 ± 28.69	2.19
Peak Adduction Velocity (Deg.S^−1^)	106.86 ± 39.85	101.46 ± 29.61	5.4
Peak Abduction Velocity (Deg.S^−1^)	−104.20 ± 18.88	−103.07 ± 29.26	1.13
**Z (+=internal /− =external)**			
Velocity at FootStrike (Deg.S^−1^)	253.64 ± 74.35	252.87 ± 74.08	0.23
Velocity at Toe-Off (Deg.S^−1^)	−43.67 ± 123.92	−43.45 ± 123.90	0.22
Peak External Rotation Velocity (Deg.S^−1^)	−255.83 ± 68.98	−254.56 ± 69.46	1.37

**Table 6:** Ankle joint velocities (means, standard deviations) from the stance limb as a function of Test and Retest anatomical co-ordinate axes (* = Significant main effect p≤0.05)

	**Test**	**Retest**	**Mean difference (Deg.s^−1^)**
**Ankle**			
**X (+ =plantar/− =dorsi)**			
Velocity at FootStrike (Deg.S^−1^)	153.18 ± 163.31	153.56 ± 163.36	0.38
Velocity at Toe-Off (Deg.S^−1^)	466.83 ± 55.41	467.83 ± 56,06	1.0
Peak Plantar Flexion Velocity (Deg.S^−1^)	739.35 ± 75.40	738.11 ± 75.74	1.24
Peak Dorsi Flexion Velocity (Deg.S^−1^)	−366.96 ± 116.45	−366.91 ± 115.68	0.05
**Y (+ =inversion/ − =eversion)**			
Velocity at FootStrike (Deg.S^−1^)	−195.08 ± 41.31	−194.45 ± 41.08	0.63
Velocity at Toe-Off (Deg.S^−1^)	180.20 ± 75.05	179.87 ± 71.69	0.33
Peak Inversion Velocity (Deg.S^−1^)	242.21 ± 66.95	240.51 ± 62.23	1.7
Peak Eversion Velocity (Deg.S^−1^)	−304.89 ± 63.17	−303.18 ± 61.95	1.71
**Z (− =internal/+ =external)**			
Velocity at FootStrike (Deg.S^−1^)	−46.14 ± 20.17	−47.69 ± 21.43	1.55
Velocity at Toe-Off (Deg.S^−1^)	−23.18 ± 68.83	−13.10 ± 69.36	10.08
Peak Internal Rotation Velocity (Deg.S^−1^)	−164.33 ± 17.19	−173.12 ± 20.15	8.79
Peak External Rotation Velocity (Deg.S^−1^)	154.44 ± 31.96	156.64 ±34.70	2.2

## References

[b1-jhk-35-15] Bell AL, Brand RA, Pedersen DR (1989). Prediction of hip joint centre location from external landmarks. Hum Mov Sci.

[b2-jhk-35-15] Besier TF, Sturnieks DL, Alderson JA, Lloyd DG (2003). Repeatability of gait data using a functional hip joint centre and a mean helical knee axis. J Biomech.

[b3-jhk-35-15] Cappozzo A, Catani F, Leardini A, Benedeti MG, Della CU (1995). Position and orientation in space of bones during movement: Anatomical frame definition and determination. Clin Biomech.

[b4-jhk-35-15] Cappozzo A (1984). Gait analysis methodology. Hum Mov Sci.

[b5-jhk-35-15] Cappozzo A, Cappello A, Croce U, Pensalfini F (1997). Surface-marker cluster design criteria for 3-D bone movement reconstruction. IEEE Transactions on Biomed Eng.

[b6-jhk-35-15] Corazza S, Mundermann L, Chaudhari AM, Demattio T, Cobelli C, Andriacchi TP (2006). A Markerless Motion Capture System to Study Musculoskeletal Biomechanics: Visual Hull and Simulated Annealing Approach. Ann Biomed Eng.

[b7-jhk-35-15] Della Croce U, Cappozzo A, Kerrigan DC (1999). Pelvis and lower limb anatomical landmark calibration precision and its propagation to bone geometry and joint angles. Medical & Biological Engineering & Computing.

[b8-jhk-35-15] Della Croce U, Leardini A, Chiari L, Cappozzo A (2005). Human movement analysis using stereophotogrammetry—part 4: assessment of anatomical landmark misplacement and its effects on joint kinematics. Gait & Posture.

[b9-jhk-35-15] Ferber R, McClay Davis I, Williams D, Laughton C (2002). A comparison of within- and between-day reliability of discrete 3D lower extremity variables in runners. J Orthop Res.

[b10-jhk-35-15] Gorton GE, Hebert DA, Gannotti ME (2009). Assessment of the kinematic variability among 12 motion analysis laboratories. Gait & Posture.

[b11-jhk-35-15] Kadaba MP, Ramakrishnan HK, Wootten ME, Gainey J, Gorton G, Cochran GV (1989). Repeatability of kinematic, kinetic, and electromyographic data in normal adult gait. J Orthop Res.

[b12-jhk-35-15] Leo T (2002). AIM Project A: CAMARC-II (computer aided movement analysis in a rehabilitation context-II). Comput Methods Programs Biomed.

[b13-jhk-35-15] McGinley JL, Baker R, Wolfe R, Morris ME (2009). The reliability of three-dimensional kinematic gait measurements: a systematic review. Gait & Posture.

[b14-jhk-35-15] Pohl MB, Chandra L, Ferber R (2010). Can the reliability of three-dimensional running kinematics be improved using functional joint methodology?. Gait & Posture.

[b15-jhk-35-15] Richards J, Thewlis D, Richards J (2008). Anatomical models and markers sets. Biomechanics in clinic and research.

[b16-jhk-35-15] Rothstein JM, Echternach JL (1993). Primer on measurement: an introductory guide to measurement issues.

[b17-jhk-35-15] Sinclair J, Taylor PJ, Edmundson CJ, Brooks D, Hobbs SJ (2012). Influence of the helical and six available cardan sequences on 3-D ankle joint kinematic parameters. Sports Biomech.

[b18-jhk-35-15] Sinclair J, Hobbs SJ, Edmundson CJ, Brooks D (2011). Evaluation of kinematic methods of identifying foot strike and toe-off during running. Int J Sp Sci and Eng.

[b19-jhk-35-15] Stacoff A, Reinschmidt C, Stüssi E (1992). The movement of the heel within a running shoe. Med Sci Sport Exer.

[b20-jhk-35-15] Thewlis D, Richards J, Bower J (2008). Discrepancies in Knee Joint Moments Using Common Anatomical Frames Defined by Different Palpable Landmarks. J App Biomech.

